# The Perfect Storm: Abnormal Baseline QT With Chronic Methadone Use and Serious Hypokalemia

**DOI:** 10.1177/23247096241286373

**Published:** 2024-10-08

**Authors:** Oscar J. Lopez, Diana Othon, Yilen K. Ng-Wong, Jose Sleiman

**Affiliations:** 1 The University of Texas Rio Grande Valley, Edinburg, USA; 2 The Ohio State University, Columbus, USA

**Keywords:** methadone, torsades de pointes, ventricular tachycardia, hypokalemia, ECG, prolonged QTc, congenital long QT

## Abstract

Methadone, a well-known drug used for pain control and as a treatment for opioid addiction, can cause arrhythmias, including torsades de pointes (TdP), which may progress to ventricular fibrillation and sudden death. We present a case of a middle-aged woman with a long history of methadone use who presented to the emergency department after experiencing cardiac arrest at home. During her hospitalization, she experienced multiple episodes of TdP that improved with isoproterenol and potassium correction. The initial diagnosis was methadone-induced prolonged QT. However, even with discontinuation of methadone, her QTc remained prolonged. Congenital long QT syndrome was suspected, and genetic testing was instructed to test in the outpatient setting. She was discharged on nadolol and a LifeVest.

## Introduction

The United States is confronted with a significant challenge as approximately 1.9 million individuals with opioid addiction, as reported by the National Institute on Drug Abuse. Over the past decade, opioid-related fatalities have remained elevated, with more than 18 000 deaths attributed to opioid overdose in 2014 alone, catapulting it to the top of the list of accidental causes of death for that year.^1^

In an endeavor to mitigate the incidence of illicit opioid overdose and associated mortalities, methadone has been employed due to its prolonged half-life (7-65 hours), a therapeutic approach initially introduced in 1965.^2^ This intervention has evidenced a reduction in overall mortality rates. Ordinarily dispensed in specialized “methadone clinics” daily, however, certain patients exhibiting adherence may be considered for home-based dosing. Of particular concern is the manifestation of QTc prolongation, likely a consequence of cardiac toxicity associated with methadone administration. This adverse effect is mechanistically attributed to the rapid blockade of the human ether-a-go-go related gene (hERG) potassium rectifier channel (Ikr), thereby impeding the cardiac rapid delayed rectifier current and altering the regulation and duration of action potentials within the ventricular myocardium.^3^

We present a case in which a 40-year-old woman on chronic methadone therapy presented with a life-threatening arrhythmia causing cardiac arrest. Once methadone was discontinued and electrolyte derangements were corrected, the patient remained with prolonged QT, which caused concern for an underlying genetic component.

## Case

A 40-year-old woman with opioid addiction, on methadone therapy (70 mg daily) for 20-plus years, was brought by the emergency medical services (EMS) after experiencing cardiac arrest.

She was found in her house after 10 minutes of cardiac arrest. She had 1 episode of pulseless electrical activity and had compressions for 15 minutes until return of spontaneous circulation was achieved. During Advanced Cardiac Life Support, she received 1 dose of epinephrine, bicarbonate, and naloxone.

**Figure 1.**
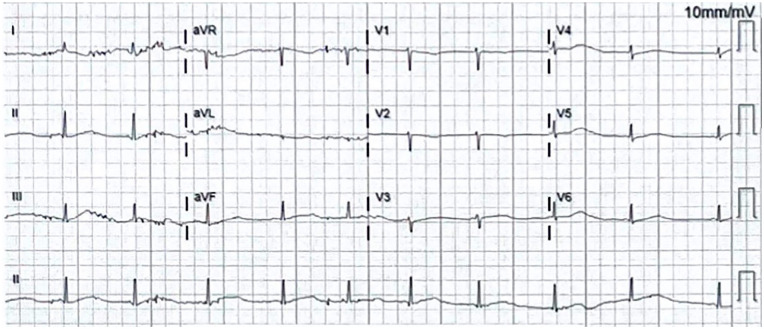
ECG on admission. Sinus bradycardia HR 55 with QT of 600, QTc 575. Demonstrates long QT syndrome’s characteristic findings: bradycardia and T-wave alternans. In this image, notched T wave in 3 leads cannot be confirmed, as there appears to be artifacts obscuring the finding.

**Figure 2.**
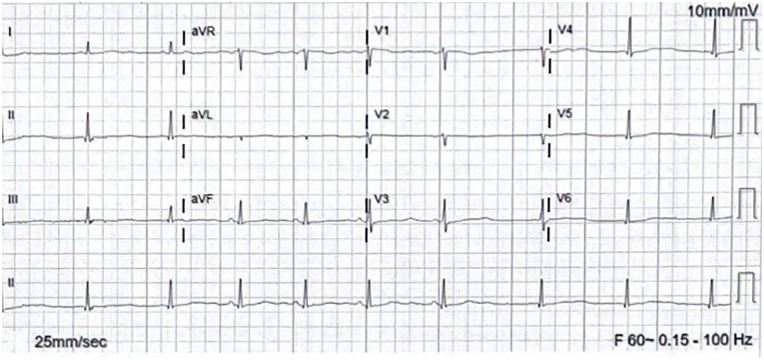
ECG on December 2023, a month prior admission. Sinus bradycardia HR 56 with QT of 673, QTc 651.

**Figure 3.**
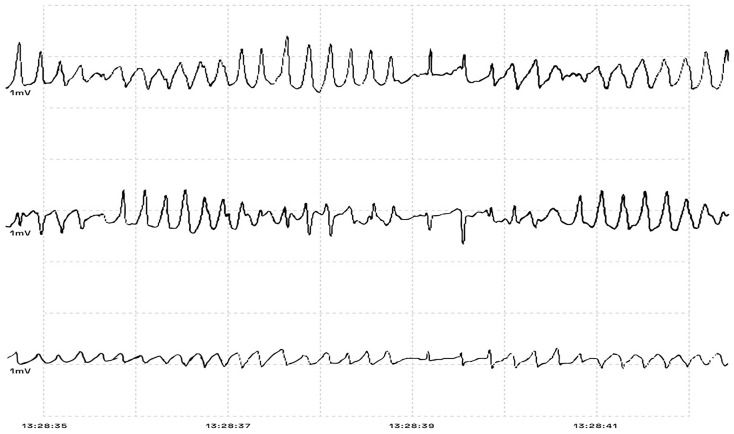
Image modified from picture taken to the cardiac monitor in the ED, depicting TdP before one of the patient’s cardiac arrest episodes.

In the emergency department (ED), her electrocardiographic (ECG) findings demonstrated a prolonged QTc ranging from 550 to 600 ms (Figure 1). A month prior to her admission, ECG showed sinus bradycardia HR 56 with QT of 673 and QTc of 651 (Figure 2). She developed several episodes of polymorphic VT/coarse VF, including some suggestive of TdP, leading to cardiac arrest (Figure 3). She was initially administered intravenous amiodarone pushes and started on the loading infusion as well as 4 g of magnesium. Amiodarone was later discontinued in the setting of increased risk of TdP, and she was optimized to lidocaine drip. Her presenting potassium was 1.9 and magnesium was normal at 2.5.

**Figure 4.**
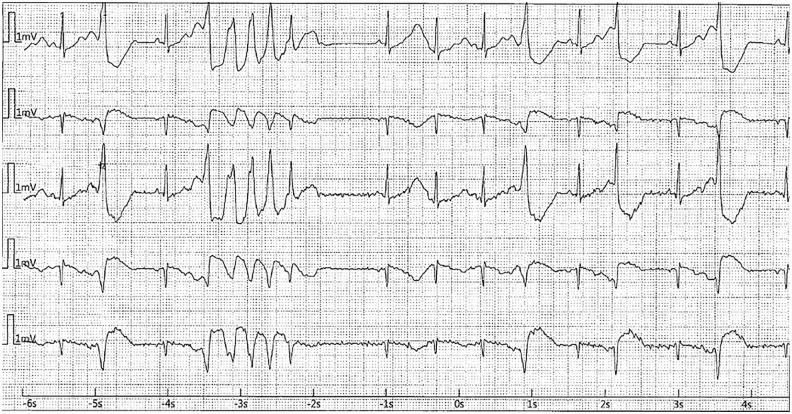
Cardiac arrest in the ICU. (Short run of TdP initiated with short-long-short sequence followed by sinus rhythm with bigeminy PVC in the setting of prolonged QTc.)

**Figure 5.**
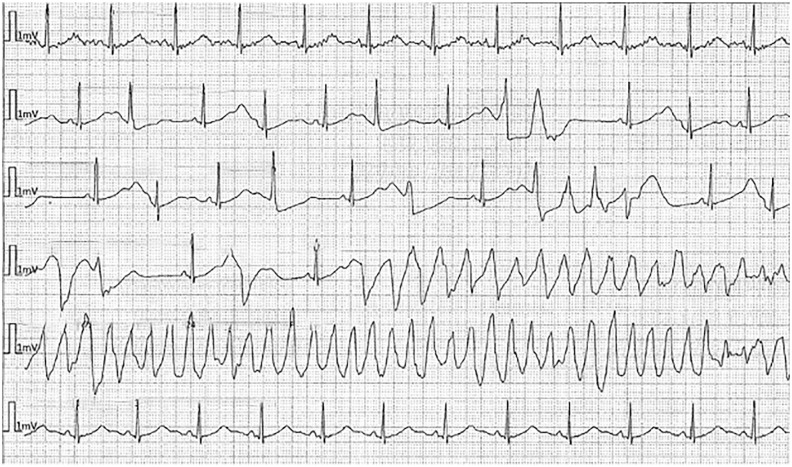
ECG during episode of cardiac arrest in the ICU.

**Figure 6.**
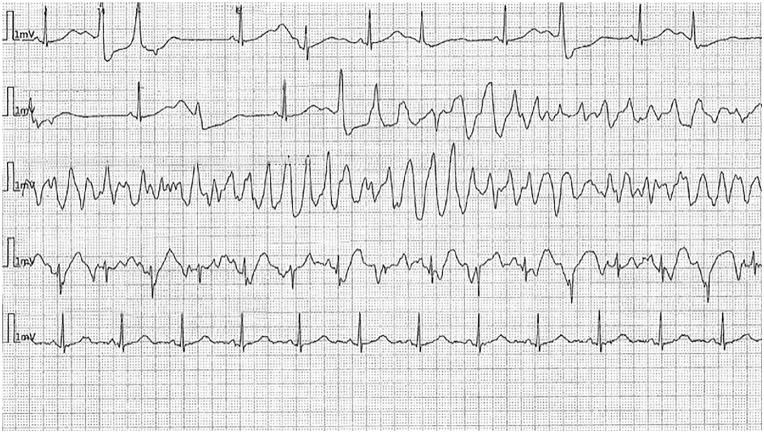
ECG in the second cardiac arrest in the ICU—Long episode of TdP initiated with short-long-short sequence in the setting on prolonged QTc.

After admission, while in the intensive care unit, she experienced several episodes of TdP initiated with a pause-dependent phenomenon in the setting of very frequent premature ventricular contractions causing short-long-short sequences (Figures 4-6). Most episodes were self-resolving but required external defibrillation in 2 instances. Lidocaine drip was used, and also isoproterenol in the setting of persistent bradycardia with a goal of heart rate above 90.

Echocardiogram was unremarkable with normal left ventricular ejection fraction of 55% to 60%. The patient was initially intubated by EMS for airway protection during the cardiac arrest and was extubated within the first 24 hours.

Neurologic examinations were done frequently, and Clinical Opioid Withdrawal Scale was performed daily. The score was ranging between 2 and 10 (no active withdrawal to mild withdrawal). Methadone 10 mg daily was prescribed as needed for withdrawal symptoms; it was given for 4 days and was discontinued 5 days after her admission.

The patient’s mental status improved during the following days; initially, she presented episodes of agitation, confusion, and hallucinations, likely secondary to opioid withdrawal and was managed appropriately. Her mentation improved considerably during hospitalization. Because potassium was consistently low, nephrology was consulted; urinary studies were under normal value, and hypokalemia was determined to be due to poor oral intake, which is a possible side effect of methadone.

**Figure 7.**
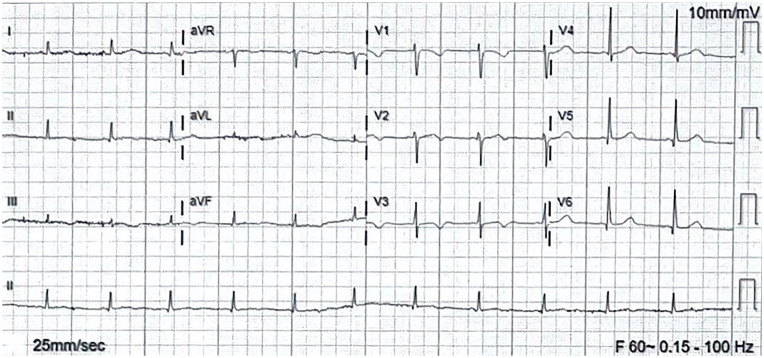
ECG before discharge. HR 75, QT 440, QTc 490.

Even with discontinuation of methadone, patient’s ECG continued to report mild prolongation of QTc of 490 ms (Figure 7), despite the absence of other QT-prolonging agents. Congenital long QT was considered, and genetic testing was discussed with the patient. Because our facility does not have the resources for genetic testing, she will follow in the outpatient setting with a cardiologist. The patient was discharged with nadolol due to concern for congenital long QT, with LifeVest and potassium per oral as well.

## Discussion

Methadone is a synthetic opioid used for the treatment of opioid use disorder. It works as a mu receptor partial agonist and physicians have used it as maintenance for opioid dependency. It has a well-known array of side effects that include nausea, vomiting, constipation, dizziness, and weight gain. It can also present with more serious side effects, such as respiratory depression. All these side effects will depend on dose, with high dose being determined as >100 mg daily.^4^ Another relatively unknown side effect is cardiotoxicity, as it has recently been associated with polymorphic ventricular tachycardia and QT prolongation, with subsequent TdP.^5^ Methadone inhibits the hERG, which is located on chromosome 7, and codes for the potassium ion channel which intercedes repolarization of the cardiac action potential.^6,7^ Methadone has also been associated with bradycardia due to its anticholinesterase effects, which can aggravate QT prolongation. Multiple papers have been published regarding this and have made QT prolongation, a known side effect of chronic methadone use.

In our patient, methadone was successfully withdrawn, and electrolytes were corrected; however, QTc was not normalized, even after several days in the hospital. Unfortunately, family history was not available as the patient had a socially complex family history. The information confirmed was the chronic methadone administration for more than 20 years, and in occasions at supratherapeutic doses. It was unknown as to how long her QT had been prolonged. This made us think about the possibility of a genetic component. Like any other genetic disease, long QT syndrome (LQTS) is mostly seen in young patients, but it has been documented in older individuals after starting a QT-prolonging medication. This medication, alongside others with similar QT prolongation effect, may cause an issue in elderly patients with underlying nondiagnosed LQTS, given the necessity for them to receive this treatment.

Long QT syndrome is a clinical diagnosis in which several factors such as symptoms, family history, and ECG findings are summarized to form the LQTS or Schwartz score.8

Long QT syndrome has been extensively evaluated over the past 4 decades, diagnosed predominantly using the Schwartz diagnostic criteria as depicted in Table 1, becoming a cornerstone in identifying this potentially life-threatening condition. The diagnosis considers 3 key components: (1) ECG findings, specifically the presence of a prolonged QTc interval in the absence of QTc-prolonging medications or other disorders; (2) clinical history, which includes symptoms such as syncope, particularly in response to triggers like stress or exercise, and congenital deafness; and (3) family history, which looks for sudden cardiac death or LQTS among first-line family members.^8,9^

**Table 1.** 1993-2013 Long QT Syndrome “Schwartz” Diagnostic Criteria.

ECG findings^a^		Points	Patient’s points
a.	QTc **≥ 480 ms** 460 – 479 ms 450 – 459 (male) ms	3 2 1	3
b.	QTc 4th minute of recovery from exercise stress test **≥ 480 ms**^b^	1	0
c.	Torsade de pointes	2	2
d.	T-wave alternans	1	1
e.	Notched T-wave in three leads	1	0
f.	Low heart rate for age^c^	0.5	0
Clinical history			
a.	Syncoped**—with stress** - without stress	2 1	0 (unknown)
b.	Congenital deafness	0.5	0 (unknown)
Family history^e^			
a.	Family members with LQTS	1	0 (unknown)
b.	Immediate family member with unexplained sudden cardiac death below age 30	0.5	0 (unknown)
**TOTAL**			**6**

SCORE:

≤ 1: low probability

1. 5 – 3: intermediate probability

*≥ 3.5: high probability*

a ECG findings in the absence of offending factors (medications or specific disorders that can cause these electrocardiographic features).

b QTc calculated with Bazett’s formula—QTc = QT/√RR.

c Low heart rate (HR) for age—resting HR below the 2nd percentile.

d Torsades and syncope must be mutually exclusive.

e Family history must include different family members for each criteria.^9^

A Schwartz score above 3.5 is considered highly suggestive of LQTS. In our patient’s case, a total score of 6 was calculated, significantly reinforcing the likelihood of this diagnosis. Notably, even in the absence of a known family history of LQTS, the patient’s clinical presentation, which included a cardiac arrest on admission, persistent hypokalemia through hospitalization, and prolonged QTc on multiple ECGs despite discontinuation of offending medication, strongly supports the diagnosis. These factors combined provide compelling evidence that the patient’s symptoms are consistent with LQTS, urging for further management and preventive measures to mitigate the risk of future cardiac events.^10^

Current literature identifies at least 17 genetic subtypes of LQTS, each characterized by mutations that affect various ion channels and proteins, specifically those related to potassium, sodium, and calcium. Among these, the most prevalent subtype is associated with a loss of function mutation in the gene KCNQ1 (LQT1), located at 11p15.5, present in around 30% to 35% of patients with LQTS.^11^ This mutation impacts the voltage-gated potassium channel Kv7.1, which is embedded within the membranes of cardiomyocytes.^11^ This channel is crucial for the activation of the delayed rectifier potassium current, which is essential for the repolarization phase of the cardiac action potential, leading to a dysfunction in this process, resulting in prolonged repolarization, and, therefore, broadening of the T wave and a prolonged QT interval on the ECG.^11^

The second most common subtype of LQTS involves a loss of function mutation in the gene KCNH2 (LQT2), located at 7q35-36, present in around 20% of patients with LQTS.9 This gene encodes the subunit Kv11.1 of the voltage-gated inward rectifying potassium channel, another critical component in cardiac repolarization.^9^ Mutations in KCNH2 similarly disrupts the normal repolarization process, resulting on a T wave that appears asymmetrical and notched, often with a low amplitude. This notched appearance is a hallmark of the delayed and irregular repolarization caused by the dysfunction of Kv11.1.9

Patients aged above 40 years have not been well represented in registries and have a lower risk for life-threatening arrhythmias than younger individuals, but still remain a considerable risk for sudden cardiac death.^12^ The TdP is suggested to occur when there are irregular heartbeats caused by premature ventricular contractions, primarily triggered by abnormal electrical activity like early after depolarization. This irregular rhythm is believed to persist due to a looping mechanism where the heart’s electrical signals get trapped in a cycle of re-entry, caused by variations in the time it takes for the heart to recover after each contraction.^13^ Beta-blocker therapy is the mainstay of management for this patient, and she was discharged with nadolol. This nonselective beta-blocker, along with propranolol, has been proven to be the most effective therapy.^14^ They are very effective in preventing arrhythmic events, even in aged patients.^15^

Furthermore, it is imperative to acknowledge that hypokalemia, particularly prevalent among women, exerts a profound influence on QTc interval prolongation. Hypokalemia precipitates repolarization abnormalities by directly inhibiting potassium channels and indirectly potentiating late sodium and calcium currents, thereby precipitating arrhythmogenic events such as TdP and other ventricular tachyarrhythmias.^16,17^

On discharge, the plan was to do genetic testing, as it was unclear which was the cause for the prolonged QT. Specific genotypes have been associated with the most common of long QT syndromes.^18^ Regarding device implantation, the role of implantable cardioverter-defibrillators (ICD) has been a topic of debate, but recent evidence recommends against use if a patient is beta-blocker naive, even after an episode of cardiac arrest.^15^ Close follow-up would be necessary given that if she develops recurrent symptoms while on beta-blocker therapy, then an ICD would be indicated. Given the uncertainty of etiology in our patient with multiple possible causes and no QT correction, it was decided to discharge with LifeVest. Medication reconciliation is a dilemma that arises in patients with this disease and this age group given the need to use QT-prolonging medications for any other medical condition. Gastrointestinal and renal diagnostic workup was performed to rule-out other causes of hypokalemia with no significant findings; genetic assessment needs to be performed, especially given high risk for TdP recurrence with her prolonged QT.

## Conclusion

Long QT syndrome is a genetic disease which predisposes patients to life-threatening arrhythmias. Most patients will present with symptoms at a young age, but it can also present in patients aged above 40 years. This age group is exposed to multiple QT-prolonging medications to treat other serious medical conditions, just like our case in which the patient was exposed to methadone for pain management. It is important to assess QT correction once this medication is stopped, to rule out any genetic component. If suspicion for LQTS is elevated, patients benefit from beta-blocker therapy, ideally nadolol or propranolol.

Guidelines have been published regarding management of LQTS and are very useful in the care of these patients. However, it can be challenging to apply these guidelines for a specific patient population, especially if their risk is intermediate and/or they have other factors which may lead to this condition. Decisions such as adequate therapy, screening, diagnosis, and ICD need are still a topic of debate. Patients should also undergo genetic testing for confirmation and further characterization of the disease. Close follow-up for symptom recurrence and medication compliance is imperative to assess for ICD need for arrhythmia prevention.
